# De novo necroptosis creates an inflammatory environment mediating tumor susceptibility to immune checkpoint inhibitors

**DOI:** 10.1038/s42003-020-01362-w

**Published:** 2020-11-04

**Authors:** Samuel T. Workenhe, Andrew Nguyen, David Bakhshinyan, Jiarun Wei, David N. Hare, Kelly L. MacNeill, Yonghong Wan, Andrew Oberst, Jonathan L. Bramson, Jalees A. Nasir, Alyssa Vito, Nader El-Sayes, Sheila K. Singh, Andrew G. McArthur, Karen L. Mossman

**Affiliations:** 1 Department of Pathobiology, Ontario Veterinary College, University of Guelph, Guelph, ON Canada; 2 McMaster Immunology Research Centre, Institute for Infectious Disease Research, Department of Pathology and Molecular Medicine, McMaster University, Hamilton, ON Canada; 3 Stem Cell and Cancer Research Institute, Department of Biochemistry and Biomedical Sciences, McMaster University, Hamilton, ON Canada; 4 Department of Immunology, University of Washington, Seattle, WA 98109 USA; 5 David Braley Centre for Antibiotic Discovery, McMaster University, Hamilton, ON Canada

**Keywords:** Cancer, Cell death and immune response, Antigen presentation, Cancer therapy

## Abstract

Cancer immunotherapies using monoclonal antibodies to block inhibitory checkpoints are showing durable remissions in many types of cancer patients, although the majority of breast cancer patients acquire little benefit. Human melanoma and lung cancer patient studies suggest that immune checkpoint inhibitors are often potent in patients that already have intratumoral T cell infiltrate; although it remains unknown what types of interventions can result in an intratumoral T cell infiltrate in breast cancer. Using non-T cell-inflamed mammary tumors, we assessed what biological processes and downstream inflammation can overcome the barriers to spontaneous T cell priming. Here we show a specific type of combination therapy, consisting of oncolytic virus and chemotherapy, activates necroptosis and limits tumor growth in autochthonous tumors. Combination therapy activates proinflammatory cytokines; intratumoral influx of myeloid cells and cytotoxic T cell infiltrate in locally treated and distant autochthonous tumors to render them susceptible to immune checkpoint inhibitors.

## Abstract (web summary)

Workenhe et al. show in mice that a combination of oncolytic HSV-1 virus and Mitomycin-C activates an inflammatory response, through necroptosis induction, that renders tumours susceptible to immune checkpoint inhibitors. These findings informs on the potential role of necroptosis in immunotherapy approaches.

## Introduction

The immune system maintains homeostasis through antigen-specific elimination of tumor cells and preventing tumor growth^1^. Successful generation of T-cell-mediated immunity involves tumor antigen presentation, a process fine-tuned by co-stimulatory/inhibitory signals and cytokines that regulate the activation of tumor-specific naive T cells to become effector T cells^2^. Various types of immunotherapies were developed through the modulation of immune regulatory mechanisms governing antitumor T cells. Of particular interest, the use of monoclonal antibodies to block inhibitory T-cell receptor signaling, collectively called immune checkpoint inhibitors (ICI), are integrated into patient standard of care to treat multiple cancer types^3–6^. However, the overall proportion of patients that respond to ICI is remarkably low owing to numerous adaptive resistance mechanisms orchestrated by tumors to defeat intrinsically developed antitumor immunity^7,8^. There is no definitive biomarker of ICI yet, although correlates such as, neoantigen load^9,10^, expression of inhibitory checkpoint ligands, interferon^11^ and proinflammatory signatures^12^, were showed to predict efficacy of ICI.

Certain types of cell death modalities emit proinflammatory signals to stimulate tumor antigen presentation and intratumoral T-cell infiltration. Cell death stimuli initiated by anthracyclines^13^, photodynamic therapy^14^, and oncolytic viruses^15–17^ elicit premortem tumor cell endoplasmic reticulum stress leading to calreticulin exposure^18^ along with release of ATP^19–21^ and high mobility group box 1^20,21^, resulting in potent antigen presentation^22^ and adaptive immunity. Although cell death inducers were extensively studied in immune-responsive transplanted tumors^13–22^, it remains largely unknown what types of combination therapies, cell death modalities, and downstream inflammatory signals can heat-up cold and immunosuppressive tumor types such as breast cancer^23,24^.

Genetically engineered mouse models of breast cancer have assisted the functional characterization of biological processes implicated in human breast cancers although they have not been extensively used to develop breast cancer immunotherapies^25,26^. Autochthonous tumors driven by overexpression of the rat oncogene *Neu* (NeuT) in BALB-NeuT mice are highly immunosuppressive and fail to spontaneously prime T cells or after immunogenic chemotherapy^23^. Consistent with previous reports^25,26^, here we report that monotherapies of chemotherapy and oncolytic virus had significant anticancer activity. The list of ICD-inducing agents is expanding although their immunomodulatory effects when applied in combination are understudied. A combined administration of oHSV-1 and Mitomycin-C extends survival of autochthonous tumor-bearing mice. This immunogenic combination therapy relies on necroptosis to activate immune-dependent anticancer effect during therapy and prophylactic vaccination. Ablation of necrosome formation in the context of therapy and dying tumor cell vaccination results in loss of efficacy. Further characterization of the tumor immune landscape shows that immunogenic therapy activates desirable inflammation and cytokine/chemokine secretion to elicit intracellular T-cell infiltration thereby rendering non-immunogenic tumors susceptible to ICI.

## Results

### A combination of oncolytic virus and chemotherapy extends survival of autochthonous tumor-bearing mice

**Fig. 1:**
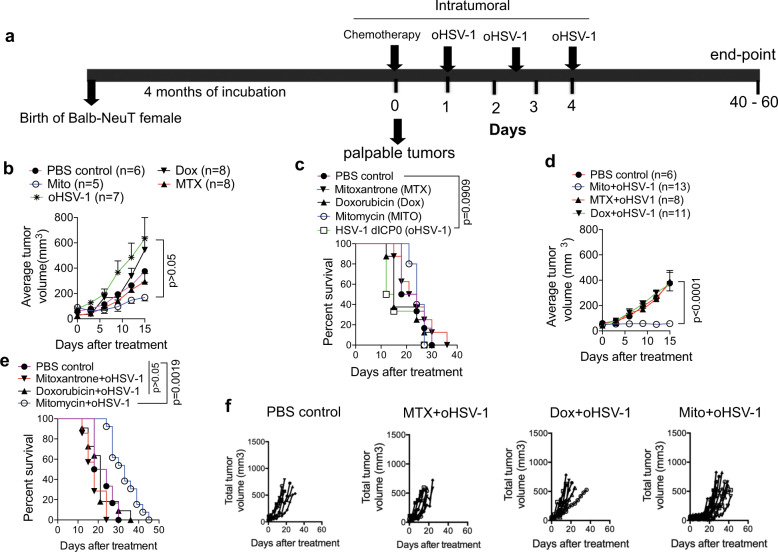
ICD-inducing monotherapies fail to limit tumor growth while combination therapy of Mito-C+oHSV-1 extends survival. **a** Outline of treatment regimens for chemotherapy and oHSV-1 in autochthonous tumors of BALB-NeuT mice. **b**, **c** Treatment with ICD-inducing monotherapies (MTX, Dox, oHSV-1) and non-ICD-inducing (Mito) neither limit tumor growth nor extend survival of tumor-bearing mice. **d**, **e** Among the various combinations of oHSV-1 and chemotherapy, Mito+oHSV-1 limits tumor growth and extends survival of tumor-bearing BALB-neuT mice. **f** Individual mice total tumor volumes as a proxy for tumor burden of BALB-NeuT mice treated with various combination therapies. Quantitative data are mean±standard deviation of total tumor volumes (Kruskal–Wallis test) **b**, **d** and Kaplan–Meier survival (Log-rank, Mantel–Cox test) **c**, **e**. All the data presented in this figure are pooled from at least two independent experiments done at different times.

We previously used TUBO cells (originally isolated from a spontaneous mammary tumor of BALB-NeuT mice^25^) in transplantation experiments to show that oHSV-1 induces markers of ICD and prolongs survival in 50% of tumor-bearing mice^27,28^. To further evaluate the systemic antitumor effects of locally applied ICD-inducing agents (oHSV-1 and chemotherapeutics) in autochthonous tumors we utilized mouse models of breast carcinogenesis driven by overexpression of the rat oncogene *Neu* (NeuT)^25,26^. Female mice that express NeuT oncogene acquire highly aggressive mammary tumors^25,26^. In all of the studies, we treated the first palpable tumor and monitored the total tumor burden arising in all of the mammary glands. Although Mitomycin-C (Mito) and Mitoxantrone (MTX) monotherapies slowed tumor growth (Fig. 1b), no monotherapy had significant tumor controlling or survival benefit on their own (Fig. 1a–c). We previously showed that in transplantable TUBO tumors combination therapy of oHSV-1 and MTX results in 69% survival of tumor-bearing mice^29^. Thus, we hypothesized that the combined administration of oHSV-1 with chemotherapeutics would provide multiple danger and pathogen signals for enhanced antitumor effect^27,29–33^. We combined oHSV-1 with ICD-inducing (MTX or Doxorubicin; Dox) or a non-ICD-inducing chemotherapy (Mitomycin-C). Administration of oHSV-1 in combination with MTX or Dox had no significant tumor controlling effect (Fig. 1d) or survival benefit (Fig. 1e). Rather the combined application of oHSV-1 with Mito had a statistically significant tumor controlling effect in BALB-NeuT mice (Fig. 1e, f).

### Necroptosis is essential for the therapeutic effect of immunogenic therapy

**Fig. 2:**
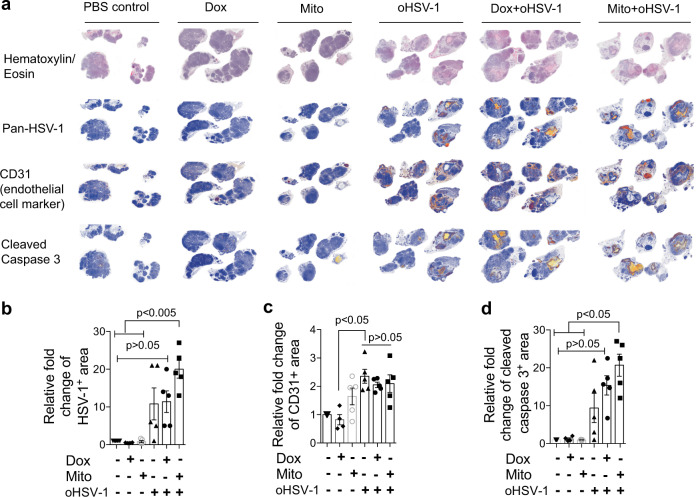
Mitomycin+oHSV-1 combination increases virus replication and tumor cell death. **a** Visualization of oHSV-1 replication (using a polyclonal antibody that detects major HSV-1 structural proteins), vascular damage (using endothelial cell marker CD31), apoptotic cell death (using cleaved caspase 3) on day 6 after start of treatment. **b**–**d** Immunohistochemistry positive signal were quantified and plotted. Quantitative data are mean±standard deviation of fold changes of positive signal relative to PBS control group. Kruskal–Wallis test with Dunn’s multiple comparison as a post hoc test was used to test statistical significance.

**Fig. 3:**
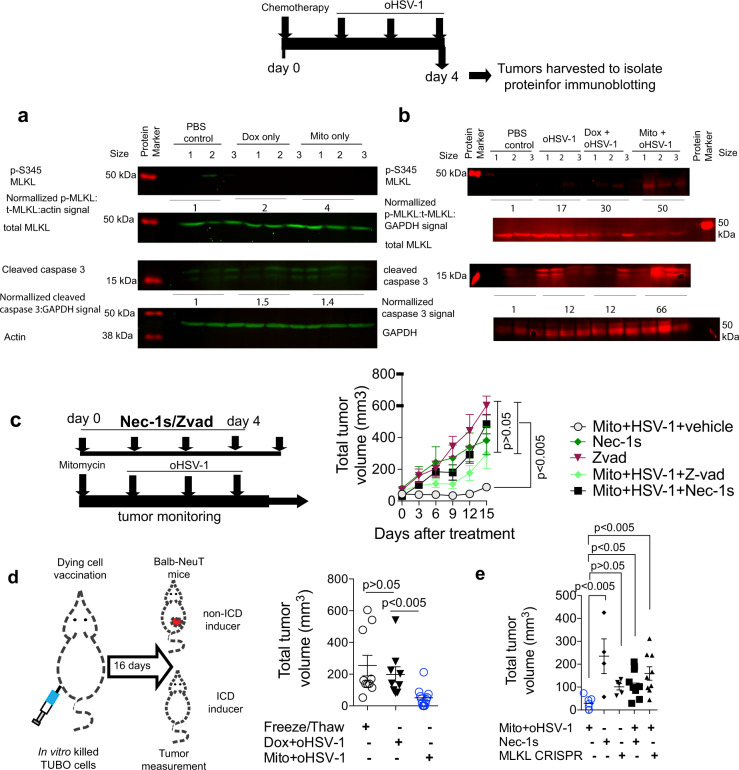
Therapeutic efficacy of immunogenic combination therapy relies on necroptosis. **a**, **b** Immunoblots of protein harvested from tumors at 96 h after start of Mito+oHSV-1 treatment show higher normalized p-MLKL and cleaved caspase 3 fluorescence intensity (*n* = 3 per treatment, labeled 1–3). Uncropped images are displayed in Supplementary Fig. 3. **c** Average total tumor volume of mice treated with Mito+oHSV-1 along with daily intraperitoneal administration of Nec-1s or Pan-Caspase inhibitor (ZVad-FMK) from day 1 to day 5. Quantitative data are mean±standard deviation from *n* = 5 mice per treatment group. **d** Average tumor burden 16 days after vaccination of naïve BALB-NeuT mice with in vitro killed TUBO cells (a cell line isolated from BALB-NeuT spontaneous tumors) (by freeze/thawing (*n* = 10) or 24 h after treatment with Mito+oHSV-1 (*n* = 12) or Dox+oHSV-1 (*n* = 9)). Dying TUBO cells were used to vaccinate 110 days old BALB-neuT mice with small palpable tumors. Quantitative data are mean±standard deviation. **e** Total tumor volume of BALB-NeuT mice 16 days after vaccination with dying wild type TUBO cells 24 h after Mito+oHSV-1 (*n* = 7), Mito+oHSV-1+Nec-1s (*n* = 9), and Nec-1s treatment (*n* = 4). Alternatively TUBO cells lacking MLKL (Supplementary fig. 4) were used for vaccination either 24 h after treatment with Mito+oHSV-1 (*n* = 9) or freeze/thawing (*n* = 5). Quantitative data are mean±standard deviation. Statistical significance of mean tumor volumes were tested using Kruskal–Wallis test with Dunn’s multiple comparison as a post hoc test.

To investigate the mechanism by which Mito+oHSV-1 exerts anticancer effect, day 6 tumors were harvested and stained for markers of virus replication, endothelial cell damage, and cell death. Monotherapies of Dox and Mito had insignificant cell death and endothelial cell damage (Fig. 2a–d). Compared with oHSV-1 and Dox+oHSV-1, Mito+oHSV-1 showed a higher level of intratumoral virus replication (Fig. 2a, b) despite in vitro addition of Mito or Dox reducing oHSV-1 replication (Supplementary fig. 1). Mito+oHSV-1 did not alter CD31 levels within tumors (Fig. 2a, c). All the monotherapies (oHSV-1, Dox, Mito) did not affect the level of apoptosis as measured by cleaved caspase 3 (Fig. 2a, d) and necroptosis as assessed by the level of p-MLKL (Fig. 3a). However, Mito+oHSV-1 therapy showed significantly higher apoptosis (Fig. 2a, d) and necroptosis (Fig. 3b, Supplementary fig. 2a, b).

Next, we evaluated which type of cell death is essential for the therapeutic effect of Mito+oHSV-1. Inhibition of caspases using a pan-caspase inhibitor partially offset the therapeutic benefit (Fig. 3c), whereas inhibition of necrosome formation using systemic Nec-1s administration^34^ significantly abolished the anticancer effect of Mito+oHSV-1 (Fig. 3c). Nec-1s+Mito+oHSV-1 treatment did not exert a statistically significant change in total lymphocyte numbers (CD4^+^ and CD8^+^) in the periphery compared with PBS control and Mito+oHSV-1 treatments (Supplementary fig. 3a, b). An established method for evaluating immune stimulating and antitumor effects of an ICD inducer is to administer in vitro killed cells as a vaccine^35,36^. TUBO cells were previously characterized murine tumor cell line established from spontaneous mammary tumors of BALB-NeuT mice^25^. BALB/NeuT mice vaccinated with dying TUBO cells treated with Mito+HSV-1, but not Dox+oHSV-1, showed significant delay in the growth of spontaneous tumors (Fig. 3d). Moreover, Nec-1s treatment or deletion of MLKL (via CRISPR/Cas9-mediated knockout; supplementary fig. S4) abrogates the antitumor effects of the vaccine (Fig. 3e). Overall, these results suggest that necroptosis is an integral part of Mito+oHSV-1 mediated immunotherapeutic effect.

### Combination therapy displays a proinflammatory cytokine signature and T-cell-mediated anticancer effect

**Fig. 4:**
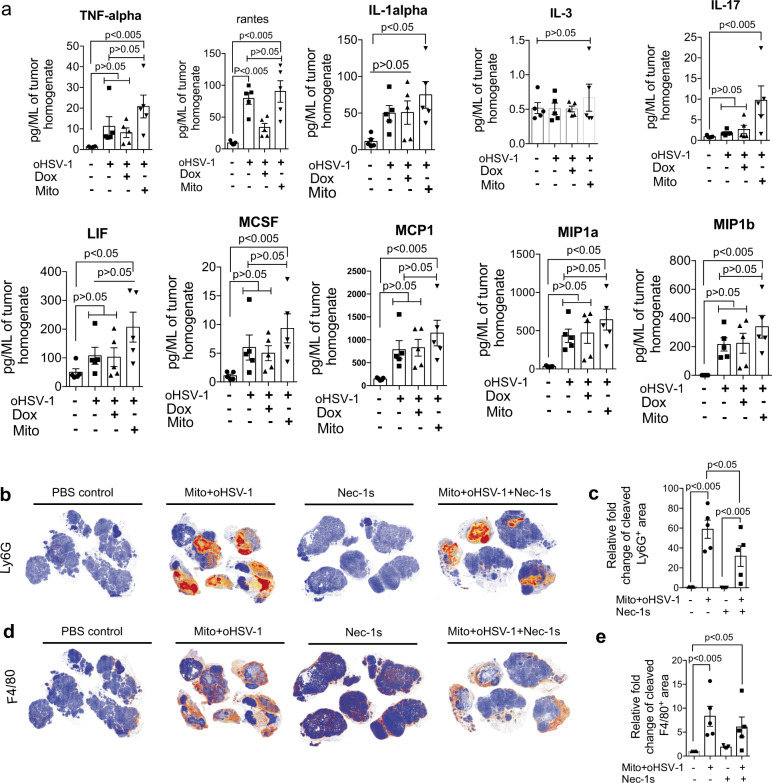
Mito+oHSV-1 shows necrosome-dependent proinflammatory signature. **a** Mito+oHSV-1 treated mammary tumors were harvested 96 h post start of treatment and tumor homogenates assayed for expression of 31 cytokines and chemokines. **b**–**e** Therapy-induced inflammation during Mito+oHSV-1 shows necrosome-dependent Ly6G+ infiltration. Autochthonous tumors of BALB-NeuT mice treated with Mito+oHSV-1 and/or Nec-1s were harvested 96 h post start of treatment and stained for Ly6G **b**, **c** and F4/80 **d**, **e**. Quantitative data are mean±standard deviation. Statistical significance was tested with one-way ANOVA or non-parametric Kruskal–Wallis test with Dunn’s multiple comparison as a post hoc test **a**, **c**, **e**. All the experiments in this figure are generated from five mice per treatment group.

To evaluate the cytokine/chemokine milieu during therapy, we analyzed the level of 31 cytokines/chemokines in tumor homogenates in treated and control mice. Untreated BALB-NeuT tumors display higher levels of immunosuppressive cytokines (IL-4 and IL-10) that are significantly downregulated after oHSV-1, Dox+oHSV-1, and Mito+oHSV-1 treatment (Supplementary fig. 5a). Other cytokines commonly upregulated by multiple therapies include Eotaxin, IL-6, and IL-15 (Supplementary fig. 5b). Cytokines/chemokines that did not show significant change include GCSF, granulocyte-macrophage colony-stimulating factor (GMCSF), IL-1b, IL-2, IL-3, IL-7, IL-9, IL-12, IP-10, CXCL-1, MIG, and VEGF (Supplementary fig. 5c). Proinflammatory cytokines (RANTES, TNF-alpha, IL-1a, IL-13, IL-17, LIF, MCSF) and chemokines (Macrophage inhibitory protein 1a (MIP1a) (CCL3), MIP1b (CCL4), Monocyte chemoattractant protein 1) (Fig. 4a) were differentially up-regulated in Mito+oHSV-1 treated tumors. Consistent with the chemokine signature, Mito+oHSV-1 treated tumors showed higher Ly6G^+^ (Fig. 4b, c) and F4/80^+^ (Fig. 4d, e) immune cell influx 96 h after start of treatment. When Nec-1s is administered along with Mito+oHSV-1, the Ly6G^+^ population is significantly reduced (Fig. 4b, c). Moreover, both Mito+oHSV-1 treated and untreated control tumors did not show intratumoral Treg infiltration (Supplementary fig. 6). Overall, these studies show that de novo necroptosis activation results in intratumoral myeloid cell infiltrate.

**Fig. 5:**
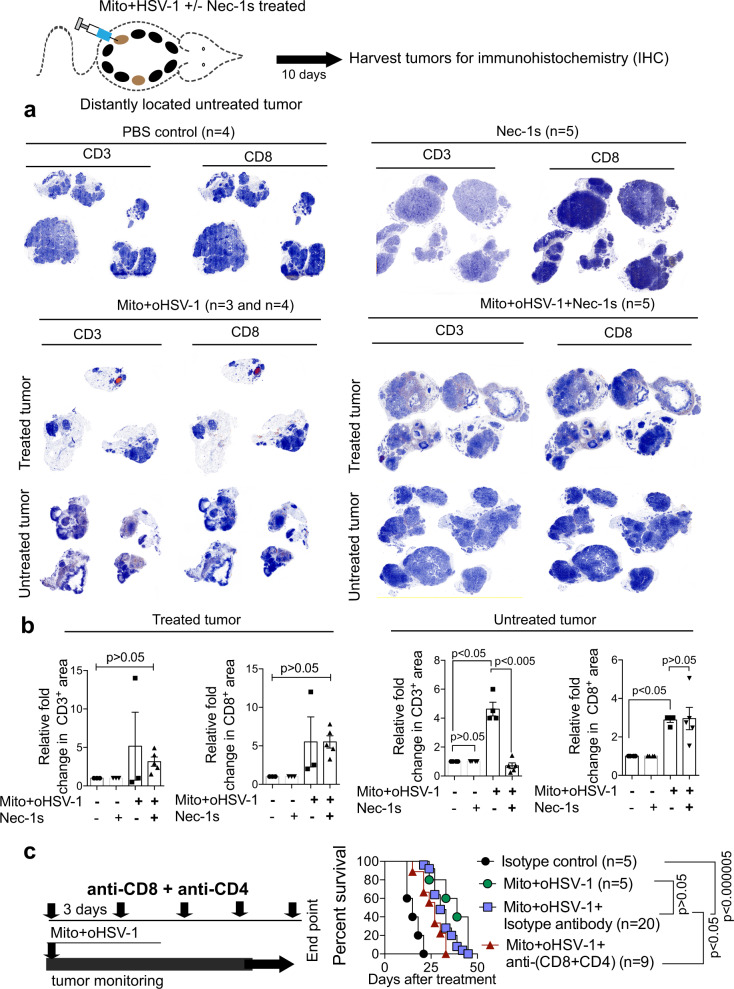
Immunogenic Mito+oHSV-1 therapy CD8+ T-cell infiltration in distant tumor lesions. **a**, **b** Ten days after Mito+oHSV-1 treatment treated and distant untreated tumor lesions were processed for IHC staining using CD3 and CD8 antibodies. Fold change in positive signal was calculated relative to the PBS controls. Quantitative data are mean±standard deviation from each treatment group (*n* = 5) (Statistical significance was tested with one-way ANOVA or non-parametric Kruskal–Wallis test with Dunn’s multiple comparison as a post hoc test). **c** BALB-neuT mice were treated with Mito+oHSV-1 along with monoclonal antibodies to deplete T cells (CD4 and CD8) or isotype control antibody. Quantitative data are Kaplan–Meier survival (Log-rank, Mantel–Cox test).

Given the inflammatory cytokine milieu and the influx of myeloid cells during Mito+oHSV-1, we hypothesized that these early events may contribute to cytotoxic T-lymphocyte infiltration within tumor lesions. To evaluate the intratumoral infiltration of CD8^+^ T cells, we isolated day 10 treated and untreated tumors (within the same mouse) for immunohistochemistry (IHC) staining of CD3/CD8 surface markers. While untreated tumors of BALB-NeuT mice lack T-cell infiltration, Mito+oHSV-1 treated and distant untreated tumors within the same mouse show higher levels of CD3^+^CD8^+^ T-cell infiltration (Fig. 5a, b). Quantification of the IHC signal shows that distant tumors are significantly infiltrated with CD3^+^CD8^+^ T cells. However, Mito+oHSV-1-treated tumors by day 10 show mostly dying tumor cells and loss of tumor architecture. As a result, the IHC quantification did not show significant T-cell infiltration in treated tumors. Inhibiting necrosome formation during Mito+oHSV-1 results in significant reduction of cytotoxic T-lymphocyte infiltration in distantly located untreated tumors (Fig. 5a, b). The abscopal effects of Mito+oHSV-1 is not an outcome of virus replication in untreated tumors, as there is no detectable virus replication in distant tumors following in vivo imaging of virus replication and IHC staining for HSV-1 structural proteins^27^ (Supplementary fig. 7).

To evaluate the importance of T lymphocytes in the therapeutic outcome of Mito+oHSV-1, we conducted survival studies in the presence or absence of CD4^+^ and CD8^+^ T cells. Depletion of T cells (CD4^+^ and CD8^+^) using monoclonal antibodies reduces the efficacy of Mito+oHSV-1 indicating that the therapeutic efficacy of Mito+oHSV-1 is T-cell mediated (Fig. 5c).

### Therapy-induced necroptosis renders autochthonous tumors susceptible to ICIs

The extent of tumor-infiltrating CD8^+^ T-cell density is significantly associated with improved clinical outcome^37^, although tumor-infiltrating CD8^+^ T cells isolated from patient tumors often display exhausted phenotypes characterized by an impaired ability to secrete effector cytokines, high expression of inhibitory receptors and altered signaling pathways^33^. Immunological interventions to block inhibitory receptors at the priming (anti-CTLA-4) and effector (anti-PD-1) stages are able to reinvigorate exhausted T cells, leading to significant therapeutic benefit in multiple human cancer types^3,4,38–40^. Human studies have found that patients harboring non-T-cell-inflamed tumors fare worse during ICI. Moreover, combined anti-CTLA-4+anti-PD-1 therapy has enhanced efficacy that exceeded either of the anti-CTLA-4 and anti-PD-1 monotherapies in melanoma patients^4^. Overall, breast cancer has low mutational load and it has not been intensively investigated for its susceptibility to clinical immunotherapies^41^. ICI using anti-CTLA-4+anti-PDL-1 is undergoing clinical evaluation in breast cancer patients^42^. ICD-inducing agents have a potential to be combined with ICI to treat immunologically cold breast cancer tumors.

**Fig. 6:**
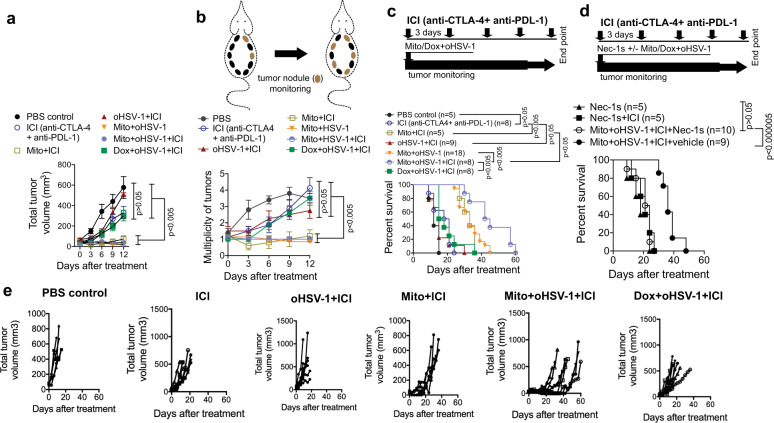
Immunogenic Mito+oHSV-1 therapy renders tumors susceptible to immune checkpoint inhibitors in necrosome-dependent manner. BALB-neuT mice treated with Mito-C+oHSV-1/Dox+oHSV-1 were concurrently treated with antibodies targeting CTLA-4 and PDL-1 (immune checkpoint inhibitor; ICI). Antitumor effect was evaluated by tumor volume reduction **a**, multiplicity of autochthonous mammary tumors **b** as well as prolonged Kaplan–Meier survival **c**. The statistical significance of tumor volume differences and multiplicity of tumors were analyzed using Kruskal–Wallis test. **d** Inhibition of necrosome using Nec-1s abrogates the efficacy of Mito+oHSV-1+ICI. Quantitative data are Kaplan–Meier survival (Log-rank, Mantel–Cox test). The statistical significance of the survival differences in **c** and **d** was analyzed using Log-rank (Mantel–Cox). **e** Individual mice total tumor volumes as a proxy for tumor burden of BALB-NeuT mice after therapy. All the data presented are pooled from at least two independent experiments done at different times.

As Mito+oHSV-1-mediated necroptosis induce systemic intratumoral cytotoxic T-lymphocyte infiltration, we hypothesized that this treatment may synergize with clinically used anti-CTLA-4+anti-PDL-1 ICI therapy^4^. Untreated tumors of BALB-NeuT mice lack intratumoral cytotoxic T lymphocytes and thus do not benefit from ICI (anti-CTLA-4 and anti-PD-L1). Moreover, Dox+oHSV-1+ICI does not show significant survival or tumor control (Fig. 6a–c, e). However, administration of ICI with Mito+oHSV-1 shows significant anticancer effect evidenced by lower tumor volume (Fig. 6a), tumor multiplicity (Fig. 6b) and prolonged survival of tumor-bearing Balb-NeuT mice (Fig. 6c). Individual mouse tumor plots of Mito+oHSV-1+ICI-treated mice show that a fraction of mice were tumor free for 40 days before relapse (Fig. 6e). Consistent with the essential role of necrosome in Mito+oHSV-1 anticancer immunity, administration of Nec-1s during the triple combination therapy abrogates the survival benefit of Mito+oHSV-1+ICI (Fig. 6d).

## Discussion

Tumors hide from immune recognition and subsequent attack by evading immune-stimulatory cell death^43^, downregulating MHC expression and secreting suppressive chemokines and cytokines^44^. As a result, cell death insults that activate premortem stress and subsequent expression of immunomodulatory molecules can be one of the ways to reinstate immunosurveillance by promoting tumor antigen uptake and presentation^45^. Using autochthonous tumors, our study demonstrates that (1) combining different classes of “bona fide” ICD-inducing agents (oncolytic virus and chemotherapy) provides a rational combinatorial therapy that activates antitumor immune response in necroptosis dependent fashion (2) immunogenic Mito+oHSV-1 therapy activates an inflammatory immune contexture associated with proinflammatory cytokine/chemokine signature and influx of myeloid cells and cytotoxic T-lymphocyte infiltrate in distant tumors, and (3) therapy-induced necroptosis is an essential aspect of Mito+oHSV-1 therapy to render tumors susceptible to ICIs.

Autochthonous tumors are highly immune suppressive and lack an intratumoral T-cell infiltrate and thus often monotherapies fail to show immunotherapeutic effect. We found out that locally administered monotherapies of Dox, Mitoxantrone, Mitomycin-C^13^, and oHSV-1^27^ fail to extend survival of autochthonous tumor-bearing mice. In support of this, a previous study showed that the anticancer effect of chemotherapies in autochthonous tumors of BALB-NeuT and conditional *K14cre*; *Cdh1*^*flox/flox*^; *Trp53*^*flox/flox*^ mice operates independent of the adaptive immune system^23^. However, both of the chemotherapies were able to show intratumoral CD3^+^ T-cell infiltrate, although the absence of lymphocytes in Rag−/− mice did not change the outcome of mammary tumorigenesis. Among the three combination therapies we tested, only Mito+oHSV-1 treatment showed adaptive immune response in a therapeutic and prophylactic vaccination setting. In support of this findings, a systemic immunogenic combination chemotherapy (cyclophosphamide–oxaliplatin) in *Kras*^*LSL-G12D/+*^*; Trp53*^*flox/flox*^ lung adenocarcinoma tumors leads to ICD-dependent CD8^+^ T-cell infiltration and synergy with ICI^45^. Collectively, these findings suggest that certain combinations of ICD-inducing therapies can exert immune-mediated anticancer effects in autochthonous tumors.

Compared with transplanted TUBO tumors^29^, autochthonous BALB-NeuT tumors are highly resistant to therapy. The median survival days and percentage of tumor-free mice after combination therapy are higher in transplanted tumors^29^ compared with autochthonous tumors in this study. These differences can be attributed to the tumor cell’s ability to sense cell death stimuli and release immunomodulatory molecules^30^, and the tumor immune landscape prior to therapy^46^. Moreover, tumor intrinsic oncogenic events may also dictate the differences in therapeutic efficacy by modulating tumor cell death signaling^47^ and influencing the tumor immune microenvironment^48^.

Depending on the models used necroptosis has immune-stimulatory^21,49,50^ as well as immunosuppressive effects^51^. During oHSV-1 monotherapy, we did not observe intratumoral necroptosis at multiple time points, although HSV-1 activates necroptosis in mouse cells while it uses its viral protein ICP6 to block necroptosis in human cells^52,53^. Our findings support that ICD-inducing properties arising from necroptosis correlates with efficacy of Mito+oHSV-1 treatment. Using Nec-1s to inhibit RIPK1-mediated necrosome formation in the context of Mito+oHSV-1 treatment leads to the loss of immunotherapeutic effect. Although Nec-1s is the preferred inhibitor of RIPK1 for in vivo use^54^, there are limitations associated with the systemic use of RIPK1 inhibitors owing to pleotropic effects of a related inhibitor Nec-1 in multiple inflammatory pathways and T-cell metabolism^54,55^. To complement experiments involving systemic Nec-1s administration, we vaccinated with dying tumor cells lacking MLKL and show that immunogenic effects of Mito+oHSV-1 treated dying cells are lost. Overall, our finding is in accordance with studies that showed antitumor immune effects of dying necroptotic cells against immunogenic tumors^21,49,50^.

Despite heterogeneity of breast cancer, targeted therapies provide durable responses with the exception of metastatic and triple negative breast cancer (TNBC)^56^. In general, breast cancer is not well infiltrated by tumor-infiltrating lymphocytes (TILs) compared with immunogenic lung and melanoma tumors. However, TNBC can display substantial immune infiltrate and patients that harbor tumors with enhanced immunomodulatory genes and/or intratumoral antitumor lymphocytes have a better prognosis^57–59^. A significant proportion of TNBC patient tumors have higher PD-L1 levels. As a result, treatment of TNBC patients with PD-1 blockade antibody (pembrolizumab) shows an overall response rate of 18.5%^60^. Use of ICI with inflammation-inducing chemotherapy and oncolytic viruses^61–68^ has gained conceptual popularity in preclinical and clinical studies^69^. This is evidenced by FDA’s recent approval of atezolizumab, a monoclonal antibody targeting PD-L1, plus chemotherapy (abraxane; nab-paclitaxel) for the treatment of adults with PD-L1-positive, unresectable, locally advanced or metastatic TNBC^69^. Moreover, different types of oncolytic viruses display additive/synergistic therapeutic efficacy when combined with ICI^61–68^ although mechanisms of synergy are not clearly identified.

Intratumoral administration of talimogene laherparepvec, a genetically engineered HSV-1 expressing GMCSF, showed higher levels of systemic and intratumoral cytotoxic T-lymphocyte infiltrate along with high PD-L1 expression in treated melanoma patient tumors^70^. Consequently, a combination of anti-PD-1 and talimogene laherparepvec therapy shows synergy to achieve 33% complete response in metastatic melanoma patients^70^. Although human trials have showed monotherapies of ICD-inducing agents can synergize with ICI^69,70^, the proportion of patients with a complete response remain quite low. It remains unknown if combining different classes of ICD inducers can be harnessed to turn on cold human tumors. Moreover, the biological processes that predict therapeutic outcome in combinatorial therapy are currently lacking. Our study provides preclinical insight that a combination of chemotherapy and oncolytic virus activates necroptosis to convert TIL-autochthonous mammary tumors into TIL+tumors that become susceptible to ICI.

In immune-responsive transplanted tumors, necroptotic fibroblasts provide adjuvant signals to facilitate immune-mediated anticancer effects^49,50,71^. Although the feasibility of vaccinating human cancer patients with necroptotic cells is not yet clear^21,49^, utilizing the immunological benefits of therapy-induced necroptosis along with ICIs may have a translational potential. In line with this, several clinically used small molecules, natural compounds and engineered oncolytic viruses that selectively induce necroptosis have a potential for synergy with ICIs^72^. Future studies should aim to characterize the immunotherapeutic potential of therapy-induced necroptosis in human breast cancer patients.

## Methods

### Study design

The aim of this study was to understand the immune-stimulatory effects of oncolytic virotherapy and various chemotherapeutics in treatment of spontaneously arising mammary tumors. We adopted an autochthonous mouse model that recapitulates a tumor model that fails to undergo spontaneous T-cell priming. We used this model to administer treatments locally at one of the tumor sites and measure systemic effect by measuring tumor volumes arising from all the 10 mammary glands.

For in vivo anticancer studies, the primary endpoint is tumor size. We have conducted several previous studies using transplantable and autochthonous tumors and determined that *n* = 5–10 is an appropriate sample size per treatment group. There were no samples or animals excluded from the analysis. For most of the experiments reported in this manuscript the efficacy of the treatments were assessed in tumor volume as a proxy for tumor growth. As a result, before animals are allocated to any treatment group their tumor volume was measured and mice were randomized to achieve equal tumor volume per treatment group.

### Cells culture

Human osteosarcoma cells (U2OS; American Type Culture Collection; ATCC, Manassas, VA) were maintained in Dulbecco’s modified Eagle’s media (DMEM) supplemented with 10% fetal bovine serum (FBS). TUBO is a cell line generated from a spontaneous mammary gland tumor of a BALB-*neu*T mouse^25^. TUBO cells are a generous gift of Dr. Guido Forni, who developed them originally. All the cell lines were mycoplasma tested and they were free of mycoplasma during the study period. TUBO cells were maintained in DMEM with 10% FBS. All media contained 2 mmol/l l-glutamine, 100U/ml penicillin, and 100 μg/ml streptomycin (Gibco). All cell lines were grown at 37 °C under humidified conditions.

### Oncolytic HSV-1

The oHSV-1 has a deletion of the entire *ICP0*-coding region and has been previously described^27^. Viruses were propagated and tittered on U2OS cells in the presence of 3 mmol/l hexamethylene bisacetamide (Sigma; St. Louis, MO). Virus purification and concentration were done using sucrose cushion ultracentrifugation^27^.

### Western blotting

Protein samples were harvested after lysing cells in radioimmunoprecipitation buffer (10 mM phosphate pH 7.4, 137 mM NaCl, 1% NP-40, 0.5% sodium deoxycholate and 0.1% sodium dodecyl sulfate) supplemented with protease inhibitor cocktail (Sigma; St. Louis, MO) and phosphatase inhibitors cocktail II and III (Sigma; St. Louis, MO). Protein extracts were resolved in 10–15% sodium dodecyl sulfate polyacrylamide gel electrophoresis and transferred to nitrocellulose membrane (Millipore, Billerica, MA). Blots were blocked in Odessey blocking buffer (LI-COR Biosciences, Lincoln, Nebraska). Western blotting detection was done after incubation with primary antibodies (anti-p-MLKL antibody, Cat#ab196436, Abcam; anti-total-MLKL, Cat#AP14272B, Abcepta; anti-cleaved caspase 3 antibody, Cat#9661, Cell Signaling Technology; and anti-beta-actin antibody, Cat#4967, Cell Signaling Technology) infrared dye-conjugated secondary antibody (Donkey-anti-Rabbit IgG-800CW conjugated, Cat#92632213, LI-COR Biosciences) using Odssey Scanner (LI-COR Biosciences, Lincoln, Nebraska).

### CRISPR knockout cell line

Design and cloning of sgRNAs targeting mouse MLKL were done as previously described^73^. In brief, the sgRNAs sequences targeting MLKL (Forward 5′-GCACACGGTTTCCTAGACGC-3′, Reverse 5′-GCGTCTAGGAAACCGTGTGC-3′) were cloned into BsmbI digested LentiCRISPR v2 plasmid (Addgene, Cambridge, MA). Single guide RNAs-Cas9 cassettes were introduced to TUBO cells by using lentivirus-mediated gene transfer. Lentivirus transduced cells were selected using 1 μg/ml Puromycin for at least 2 weeks and depletion of the protein was verified by immunoblotting.

### Treatment with chemotherapeutics and oHSV-1

Mice were maintained at the McMaster University Central Animal Facility and all procedures were performed in full compliance with the Canadian Council on Animal Care and approved by the Animal Research Ethics Board of McMaster University. Weaned mice were genotyped for the presence of the transgenes. Transgenic mice (BALB-NeuT, Gp/PyMT, FVB-NeuT) were allowed to age for 110 days or until the mammary glands start to develop palpable tumors. In the entire study, tumor measurements and treatments were initiated once the first tumor reached palpable size. Chemotherapeutics were administered once and in the combination setting, chemotherapeutics were administered 1 day prior to intratumoral oncolytic virus treatment (three doses of 2 × 10^7^ pfu^27^). Chemotherapeutic doses were 860 mM (1.25 mg/Kg) Dox hydrochloride (Sigma; St. Louis, MO), 6 mM Mito-C (4 mg/Kg) (Sigma; St. Louis, MO) or 5 μM MTX (Sigma; St. Louis, MO) (6.5 mg/Kg)^13,74^, administered intratumorally in 50 μl volume. The RIPK1 inhibitor (Nec-1s) (BioVision, Milpitas, CA) was administered at a dose of 6 mg/kg. The pan-caspase inhibitor ZVAD-FMK (Minneapolis, MN) was used at a dose of 10 mg/kg. Inhibitors or the 5% dimethyl sulphoxide vehicle control were administered intraperitoneally every day for 4 days of Mito+oHSV-1 treatment. All tumors arising from the 10 mammary glands were measured every three days until mice reach endpoint. Mice having a total tumor volume above 525 mm^3^ were classified as endpoint and sacrificed^29^.

### Vaccination with dying TUBO cells

BALB-*Neu*T mice were vaccinated with dying TUBO cells. The control group was vaccinated with TUBO cells that were freeze-thawed. TUBO cells were infected with HSV-1 dICP0 at MOI of 3 for 1 h and then add back media with 25 μM Dox or 30 μM Mito-C was applied^29^. Nec-1s was used in the context of Mito+oHSV-1 treatment at 100 μM. Cells were incubated for 24 hr before scrapping to pellet the cells for vaccination. Each mouse was vaccinated with three million dying cells. Mammary glands were palpated and measured every 3 days.

### Immunohistochemistry

Treated and control mammary tumors were fixed in 10% formalin for 48 h and then transferred to 70% ethanol until histological processing. Tumor sections were processed for Hematoxyilin–Eosin staining or immunohistochemistry using antibodies listed in Supplementary Table 1. All the antibodies used in this study were validated by the supplier as well as independent publications from academic laboratories. However, before we use any of the antibodies, we have validated their suitability by titrating the amount of antibody and running positive and negative controls. All antibodies were stained on the Leica Bond RX Automated Stainer with either Epitope Retrieval Buffer 1 (ER1), Epitope Retrieval Buffer 2 (ER2), or with Enzyme 1 (Leica). All antibodies were diluted in Power Vision Super blocker (Leica). The IHC slides were scanned using Aperio ScanScope slide scanner (Aperio Technologies, CA), and the images were analyzed using Positivity Pixel Count 9.0 algorithm within ImageScope software (Aperio Technologies, CA) as described previously^75^.

### Tumor protein isolation

Mice were anaesthetized and euthanized before resection of the tumors. Tumors were cut into small pieces and homogenized in the presence of tissue extraction solution (50 mM Tris, pH 7.4, 250 mM NaCl, 5 mM EDTA 2 mM Na3VO4, 1 mM NaF, 20 mM Na4P2O7, 1 mM beta-glycerophosphate, 1% NP-40). Homogenized tumors were incubated on ice for 30 min. Whole-tumor lysates were clarified by two sequential centrifugations at 13,000 × *g* for 10 min at 4 °C. Twenty to eighty micrograms of total protein was used for western blot analysis.

### Cytokine analysis

Cytokine analysis was carried out on tumor homogenates harvested using tissue extraction buffer. Tumor homogenates with equal amounts of protein concentration were shipped to Eve Technologies (Calgary, Alberta, Canada) for 31-Plex murine cytokine/chemokine analysis.

### Antibodies

The immune checkpoint blockade antibodies anti-CTLA-4 (clone 9H10, BioXcell), and anti-PDL-1 (clone 10 F.9G2, BioXcell) were used alone or in combination with HSV-1+Mito-C. For ICI treatments, mice with palpable tumors volume below 100 mm^3^ were injected with 200 μg of the antibodies every 3 days. In the combination treatments ICI antibodies were applied at the same day as the start of HSV-1 treatment. Treatments were continued until mice reached end point. Monoclonal antibodies targeting CD4 (clone GK1.5, BioXcell) and CD8 (Clone 2.43, BioXcell) T cells were administered intraperitoneally at a dose of 250 μg starting 2 days before treatment and applied every other day for the first two weeks and once a week thereafter. Depletion of cells after antibody administration was verified by flow cytometry.

### Statistics and reproducibility

BALB-NeuT female mice tumors take a minimum of 110 days to become palpable and the incidence of macroscopic tumors is a stochastic event making larger experiments difficult to be synchronized. As a result, we conducted experiments with less than five mice per treatment group (while including multiple treatment and controls groups) and pooled experimental results collected from multiple independent experiments.

For each statistical analysis used, normality of the distributions and equality of variance assumptions were tested before running the statistical analyses. One-way analysis of variance, non-parametric Kruskal–Wallis test and *t* test were used to determine the statistical significance of the differences in means. For analyzing the statistical significance of the difference in Kaplan–Meier survival between treatments, the Log-rank (Mantel–Cox) test was used. All the tests were two-sided. The null hypothesis was rejected for *p* values <0.05. All data analyses were carried out using GraphPad Prism (La Jolla, CA, USA).

### Reporting summary

Further information on research design is available in the Nature Research Reporting Summary linked to this article.

## Supplementary information

Supplementary Information

Description of Additional Supplementary Files

Supplementary Data 1

Reporting Summary

## Data Availability

The source data behind the graphs in this paper are available in Supplementary Data 1. All other data are available from the authors upon reasonable request.
