# Supplementary material for: De novo necroptosis creates an inflammatory environment mediating tumor susceptibility to immune checkpoint inhibitors
Source: Commun Biol. 2020 Nov 4;3:645. doi: 10.1038/s42003-020-01362-w (PMC7643076; doi:10.1038/s42003-020-01362-w)
Supplement: Supplementary file 1 — Supplementary Information [file 42003_2020_1362_MOESM1_ESM.pdf]

Supplementary Figure 1

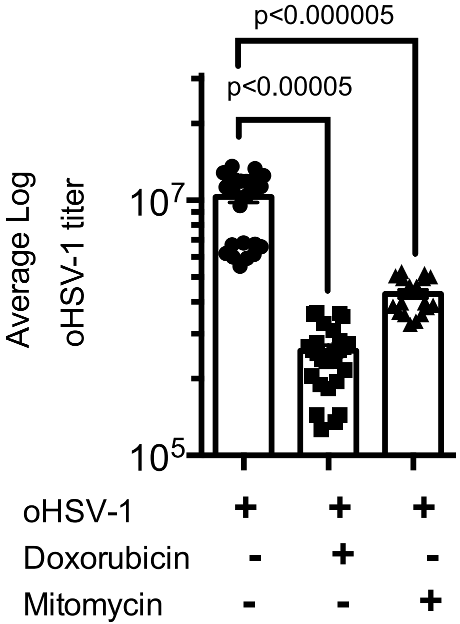

Supplementary Figure 1. TUBO cells were infected with oncolytic HSV-1 in the presence or absence of Mito and Dox and the amount of virus yield was quantified two days post infection by virus titration. Quantitative data are mean ±standard deviation of virus titer analyzed for significance using Kruskal-Wallis test.

Supplementary Figure 2

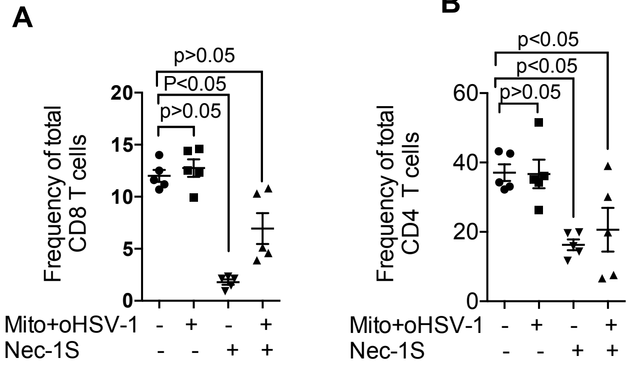

Supplementary Figure 2. Balb-NeuT mouse tumors were treated with Mito+oHSV-1 with/without Nec-1s and peripheral blood was collected five days after start of treatment for FACS based analysis of CD8+ (a) and CD4+ (b) T lymphocyte numbers. Quantitative data are mean ±standard deviation of lymphocyte frequency analyzed for statistical significance using Chi-Square test.

Supplementary Figure 3

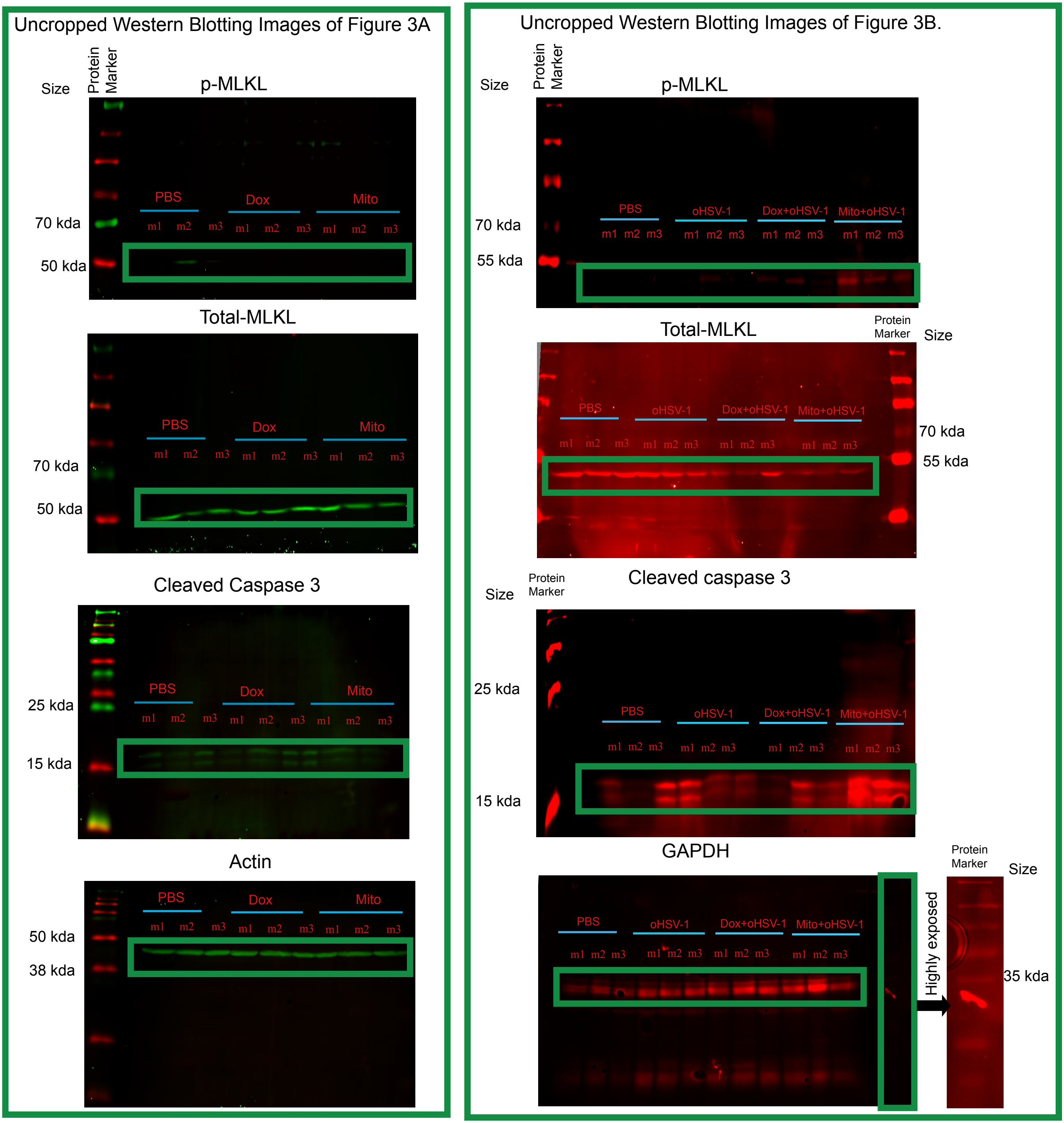

Supplementary Figure 3. Immuno blots of protein harvested from tumors at 96 hour after start of Mito+oHSV-1 treatment show higher normalized p-MLKL and cleaved caspase 3 fluorescence intensity (n=3 per treatment, labeled 1-3). Cropped images are displayed in Figure 3 **a** and **b.**

Supplementary Figure 4

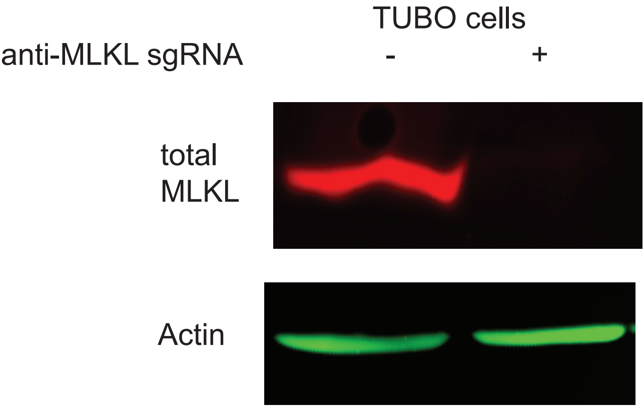

Supplementary Figure 4. Western blotting showing CRISPR/Cas9 mediated knockout of MLKL.

Supplementary Figure 5

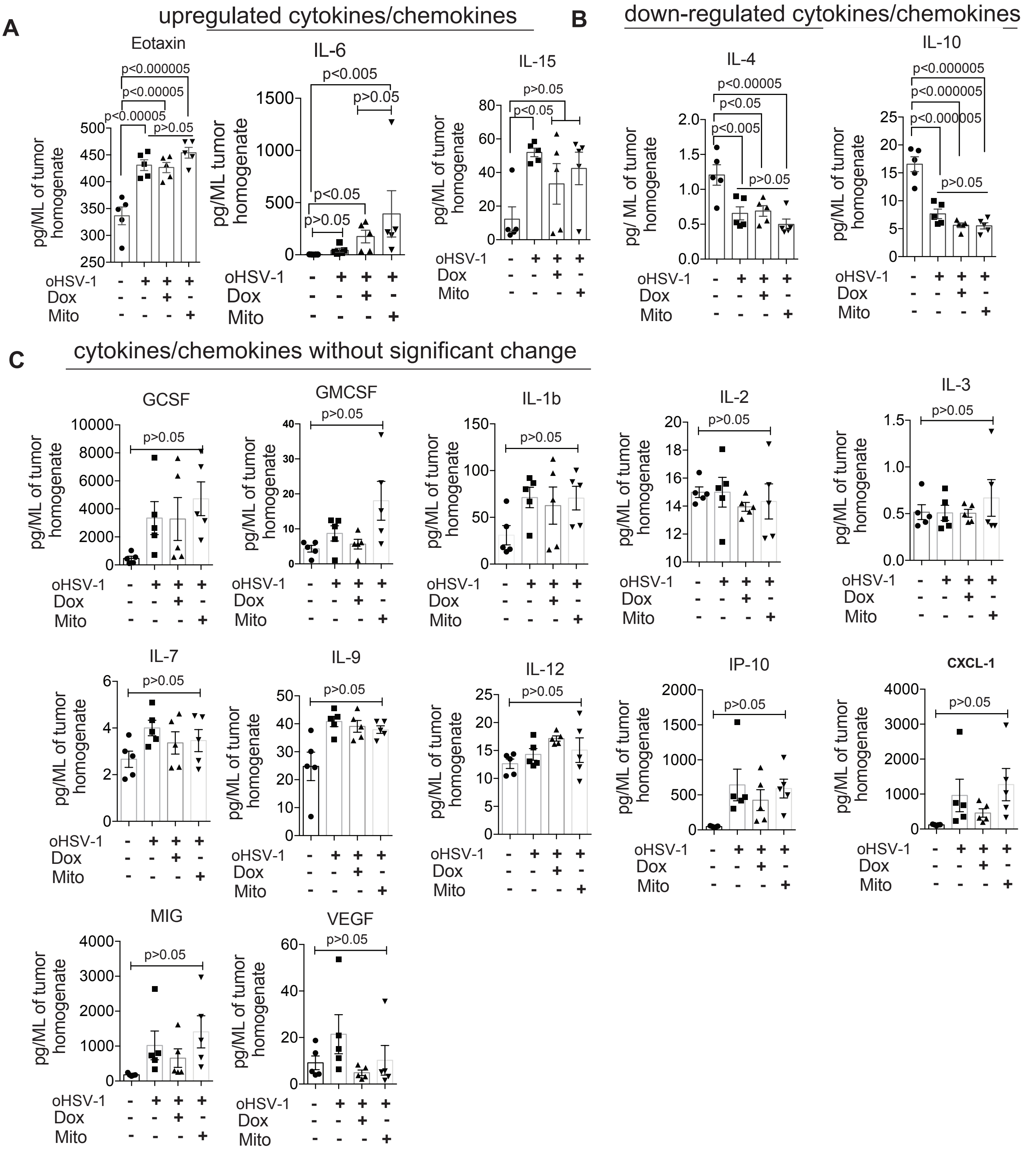

Supplementary figure 5. a-c Cytokine/chemokine levels in tumor homogenates harvested from treated and control BALB-NeuT mice. Homogenized tumor samples harvested 96 hours post treatment were analyzed by Eve Technologies. Quantitative data are mean ±standard deviation of cytokines/chemokine measurements (n=5 per treatment group) and analyzed for statistical significance using Kruskal-Wallis test.

Supplementary Figure 6

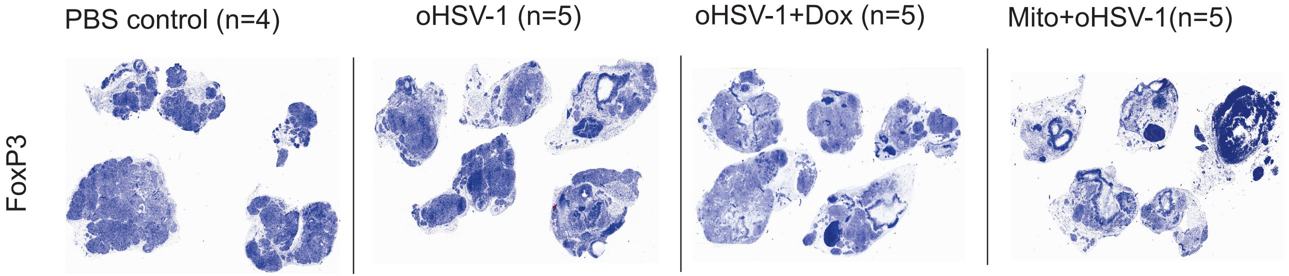

Supplementary Figure 6. FOXP3+ immunohistochemical staining 6 days after treatment of autochthonous tumors of BALB-NeuT mice.

Supplementary Figure 7

A

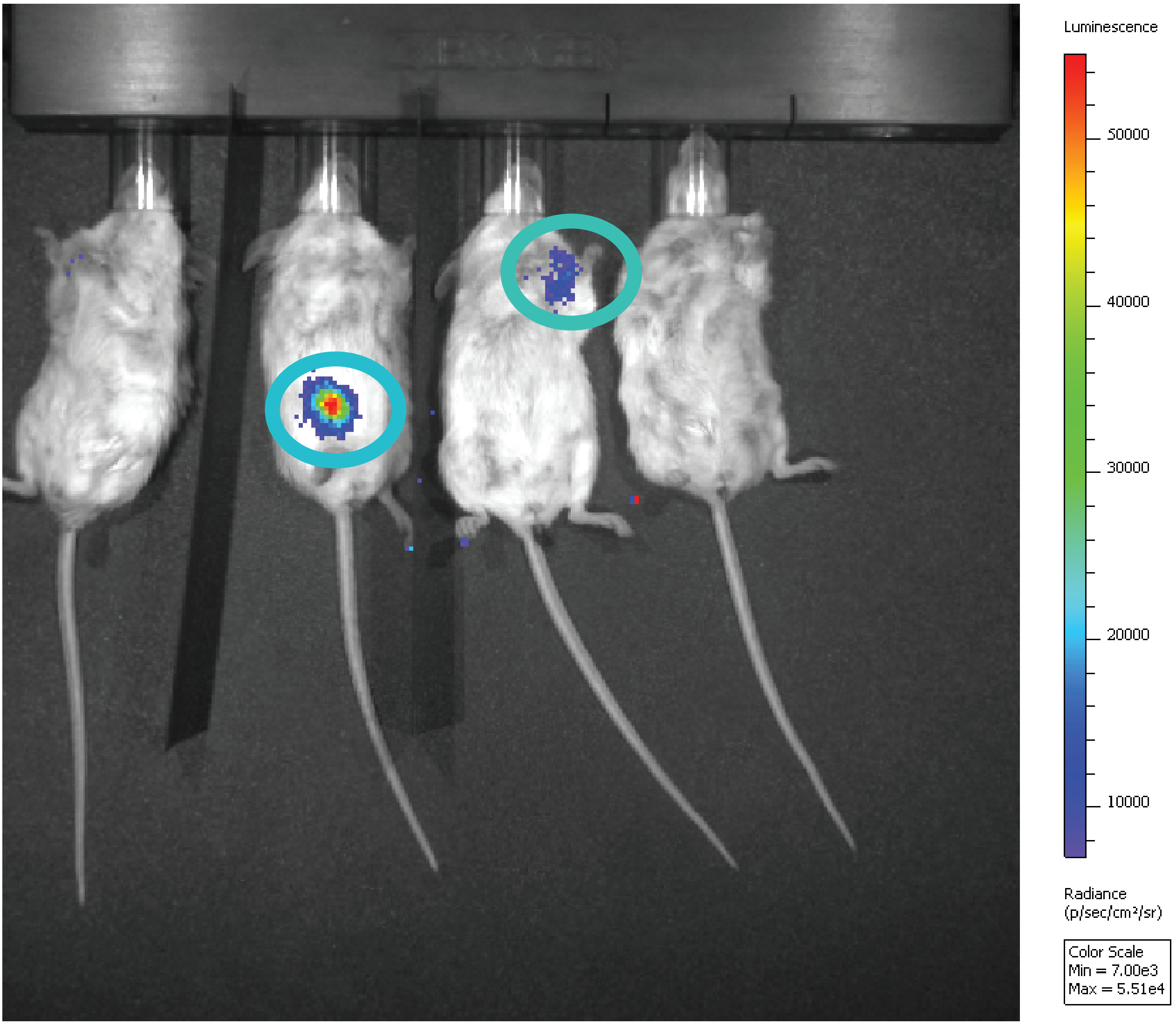

B

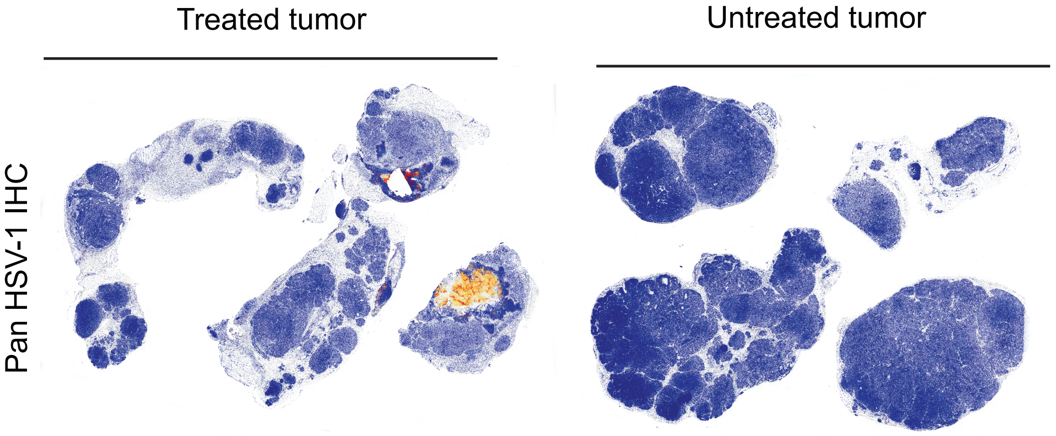

Supplementary Figure 7. a Imaging of replicating virus in BALB-NeuT mice six days after oHSV-1 treatment using the In vivo Imaging Spectrum (IVIS). Blue circles highlight tumors with evidence of virus replication. **b** Locally treated (with oncolytic HSV-1) and distantly located untreated tumors within the same mouse were harvested 6 days post treatment and after IVIS imaging for immunohistochemical detection of HSV-1 glycoproteins.

Supplementary Table 1. Immunohistochemistry antibodies, retrieval method and detection.

Antibody	Commercial provider	Catalogue number	Primary antibody dilution	Antigen Retrieval method	Detection system
Pan-HSV-1	DAKO	B0114	1:2000	ER-1 Low pH retrieval buffer - 20 minutes	Bond refine detection kit (no post primary)
Cleaved Caspase 3	Cell Signaling Technology	9664	1:400	ER2 High pH retrieval buffer – 20 minutes	Bond refine detection kit (no post primary)
Foxp3	EBio-Affymetrix	14-5773-82	1:100	Epitope retrieval (ER) 1 (20min)	Vector B4001 Rabbit anti-Rat (mouse absorbed) at 1:100 and Bond refine detection kit (no post primary)
F4/80	AbD Sertec	MCA497R	1:500	ER 1 (10min)	Vector B4001 Rabbit anti-Rat (mouse absorbed) at 1:100 and Bond refine detection kit (no post primary)
Ly6G	BioLegend	127602	1:1000	ER 2 (20 min)	Vector B4001 Rabbit anti-Rat (mouse absorbed) at 1:100 and Bond refine detection kit (no post primary)
CD4	EBio Affymetrix	14-9766	1:800	ER 2(20 min)	Vector B4001 Rabbit anti-Rat (mouse absorbed) at 1:100 and Bond refine detection kit (no post primary)
Cd8a	EBio-Affymetrix	14-0808	1:1000	ER 2 (20min)	Vector B4001 Rabbit anti-Rat (mouse absorbed) at 1:100 and Bond refine detection kit (no post primary)
CD31	Histobiotec (DiaNova)	Dia-310	1:60	ER1 (10 min)	Vector B4001 Rabbit anti-Rat (mouse absorbed) at 1:100 and Bond refine detection kit (no post

					primary)
CD3	Abcam	Ab16669	1:50	ER2 (20 min)	Bond refine detection kit (no post primary)
