# Supplementary material for: De novo necroptosis creates an inflammatory environment mediating tumor susceptibility to immune checkpoint inhibitors
Source: Commun Biol. 2020 Nov 4;3:645. doi: 10.1038/s42003-020-01362-w (PMC7643076; doi:10.1038/s42003-020-01362-w)
Supplement: Supplementary file 2 — Description of Additional Supplementary Files [file 42003_2020_1362_MOESM2_ESM.pdf]

Description of Additional Supplementary Files

File Name: Supplementary Data 1
Description: Data used to construct main figures.
