# Supplementary material for: De novo necroptosis creates an inflammatory environment mediating tumor susceptibility to immune checkpoint inhibitors
Source: Commun Biol. 2020 Nov 4;3:645. doi: 10.1038/s42003-020-01362-w (PMC7643076; doi:10.1038/s42003-020-01362-w)
Supplement: Supplementary file 4 — Reporting Summary [file 42003_2020_1362_MOESM4_ESM.pdf]

nature research

Corresponding author(s): Workenhe Samuel

Last updated by author(s): 2020/09/17

Reporting Summary

Nature Research wishes to improve the reproducibility of the work that we publish. This form provides structure for consistency and transparency in reporting. For further information on Nature Research policies, see Authors & Referees and the Editorial Policy Checklist .

Statistics

For all statistical analyses, confirm that the following items are present in the figure legend, table legend, main text, or Methods section.

n/a	Confirmed	
☐	☒	The exact sample size (*n*) for each experimental group/condition, given as a discrete number and unit of measurement
☐	☒	A statement on whether measurements were taken from distinct samples or whether the same sample was measured repeatedly
☐	☒	The statistical test(s) used AND whether they are one- or two-sided<br>*Only common tests should be described solely by name; describe more complex techniques in the Methods section.*
☐	☒	A description of all covariates tested
☐	☒	A description of any assumptions or corrections, such as tests of normality and adjustment for multiple comparisons
☐	☒	A full description of the statistical parameters including central tendency (e.g. means) or other basic estimates (e.g. regression coefficient) AND variation (e.g. standard deviation) or associated estimates of uncertainty (e.g. confidence intervals)
☐	☒	For null hypothesis testing, the test statistic (e.g. *F*, *t*, *r*) with confidence intervals, effect sizes, degrees of freedom and *P* value noted<br>*Give P values as exact values whenever suitable.*
☒	☐	For Bayesian analysis, information on the choice of priors and Markov chain Monte Carlo settings
☒	☐	For hierarchical and complex designs, identification of the appropriate level for tests and full reporting of outcomes
☐	☒	Estimates of effect sizes (e.g. Cohen's *d*, Pearson's *r*), indicating how they were calculated

Our web collection on statistics for biologists contains articles on many of the points above.

Software and code

Policy information about availability of computer code

Data collection	no codes were used for data collection
Data analysis	Data analysis was done by using GraphPad Prism (La Jolla, CA, USA)

For manuscripts utilizing custom algorithms or software that are central to the research but not yet described in published literature, software must be made available to editors/reviewers. We strongly encourage code deposition in a community repository (e.g. GitHub). See the Nature Research guidelines for submitting code & software for further information.

Data

Policy information about availability of data

All manuscripts must include a data availability statement. This statement should provide the following information, where applicable:

- Accession codes, unique identifiers, or web links for publicly available datasets
- A list of figures that have associated raw data
- A description of any restrictions on data availability

The source data behind the graphs in this paper are available in Supplementary Data 1. All other data are available from the authors upon reasonable request.

Field-specific reporting

Please select the one below that is the best fit for your research. If you are not sure, read the appropriate sections before making your selection.

☒ Life sciences ☐ Behavioural & social sciences ☐ Ecological, evolutionary & environmental sciences

Life sciences study design

All studies must disclose on these points even when the disclosure is negative.

Sample size	For in vivo anticancer studies, the primary endpoint is tumor size. We have conducted several previous studies using transplantable and autochthonous tumors and determined that n=5-10 is an appropriate sample size per treatment group. A sample size of 10 mice per group will achieve 90% power to detect an effect size of 0.4, using a one-way ANOVA and at a significance level of 0.05. The effect size is defined as the variance of the means divided by the within group variance. nQuery Advisor 7.0 was used for the sample size/power calculation.
Data exclusions	Not Applicable (There were no samples or animals that were excluded from the analysis)
Replication	In the majority of the experiments reported data were a result of at least three independent times conducted at three different times. In some in vivo studies experiments were repeated experiments only twice.
Randomization	Tumor bearing animals had a small variability in tumor volume. A day before start of treatment tumor volumes were measured and mice were randomized to have an equal average tumor volume per treatment group. This is described in our previous publications cited in Materials and methods (in vivo mouse tumor studies). For this study our treatment effect is tumor volume. As a result before animals are allocated to any treatment group their tumor volume was measured and mice were randomized to achieve equal tumor volume per treatment group.
Blinding	blinding is useful to prevent experimental artifacts that could be caused by knowledge of the subjects. It prevents the investigator doing the study not to be subconsciously influenced to see something consistent with a hypothesis. However, for this study we did not do blinding.

Reporting for specific materials, systems and methods

We require information from authors about some types of materials, experimental systems and methods used in many studies. Here, indicate whether each material, system or method listed is relevant to your study. If you are not sure if a list item applies to your research, read the appropriate section before selecting a response.

Materials & experimental systems

n/a	Involved in the study	
☐	☒	Antibodies
☐	☒	Eukaryotic cell lines
☒	☐	Palaeontology
☐	☒	Animals and other organisms
☒	☐	Human research participants
☒	☐	Clinical data

Methods

n/a	Involved in the study	
☒	☐	ChIP-seq
☐	☒	Flow cytometry
☒	☐	MRI-based neuroimaging

Antibodies

Antibodies used	All the antibodies used in the study are described in the methods section and supplementary Table 1.
Validation	All the antibodies used in this study were validated by the supplier as well as independent publications from academic labs. However, before we use any of the antibodies, we have validated their suitability by titrating the amount of antibody and running positive and negative controls.

Eukaryotic cell lines

Policy information about cell lines

Cell line source(s)	TUBO
Authentication	we received TUBO cells from Dr. Guido Forni who developed them originally however we did not authenticate the cell lines.
Mycoplasma contamination	All the cell lines were mycoplasma tested and they were free of mycoplasma during the study period.
Commonly misidentified lines (See ICLAC register)	none

Animals and other organisms

Policy information about studies involving animals; ARRIVE guidelines recommended for reporting animal research

Laboratory animals	we used Balb-NeuT mice in Balb-C background at the age of 110 days where tumors are palpable.

Wild animals | no wild animals were used in this study

Field-collected samples | no field collected samples were used

Ethics oversight | Ethics approval was done by the Centralized Animal Facility Ethics Committee

Note that full information on the approval of the study protocol must also be provided in the manuscript.

Flow Cytometry

Plots

Confirm that:

- [x] The axis labels state the marker and fluorochrome used (e.g. CD4-FITC).
- [x] The axis scales are clearly visible. Include numbers along axes only for bottom left plot of group (a 'group' is an analysis of identical markers).
- [x] All plots are contour plots with outliers or pseudocolor plots.
- [x] A numerical value for number of cells or percentage (with statistics) is provided.

Methodology

Sample preparation	Immune cells were collected from enzymatically dissociated tumor cell suspensions.
Instrument	BD LSR Fortessa cytometer (BD Biosciences)
Software	FlowJo (TreeStar)
Cell population abundance	we did not conduct cell sorting experiments
Gating strategy	Gating strategy and classification of positive and negative populations were done by using Florescent Minus One Controls.

- [x] Tick this box to confirm that a figure exemplifying the gating strategy is provided in the Supplementary Information.
